# Clinical Characteristics and Molecular Epidemiology of Noroviruses in Outpatient Children with Acute Gastroenteritis in Huzhou of China

**DOI:** 10.1371/journal.pone.0127596

**Published:** 2015-05-26

**Authors:** Weihua Zou, Dawei Cui, Xiang Wang, Huihui Guo, Xing Yao, Miao Jin, Qiuling Huang, Min Gao, Xiaohong Wen

**Affiliations:** 1 Department of Clinical Laboratory, Huzhou Central Hospital, Huzhou 313000, China; 2 Center of Clinical Laboratory, The First Affiliated Hospital, School of Medicine, Zhejiang University, Hangzhou, 310003, China; 3 Center of Clinical Laboratory, The First People’s Hospital of Huzhou, Huzhou Teachers College, Huzhou, 313000, China; 4 National Institute for Viral Disease Control and Prevention, Chinese Center for Disease Control and Prevention, Beijing, 102206, China; Kliniken der Stadt Köln gGmbH, GERMANY

## Abstract

**Background:**

Noroviruses (NoVs) are considered major causative pathogens associated with the morbidity and mortality of young children with acute gastroenteritis. However, few studies have examined NoVs causing acute diarrhea among outpatient children worldwide. This study was conducted to investigate the clinical features and molecular epidemiology of NoVs in outpatient children with acute gastroenteritis in Huzhou, China, between April 2013 and April 2014.

**Methods:**

Stool specimens from 1346 outpatient children enrolled (under 5 years of age) with acute gastroenteritis were examined for NoVs by multiplex RT-PCR, and sequences of the partial capsids of NoVs were analyzed phylogenetically, while the relevant clinical data were analyzed statistically.

**Results:**

Of 1346 specimens, 383 (28.5%, 383/1346) were positive for NoVs. The proportion of GII genotypes (26.9%) was significantly higher than that of GI genotypes (1.6%). The GII.4 genotype was the most prevalent of GII genotypes and was clustered into GII.4/Sydney (37.8%) and GII.4/2006b (62.2%), whereas GI strains were clustered into GI.1. Additionally, the younger children (12 to <24 months of age) were more susceptible to NoVs than children in other age groups, and the highest percentage of NoV infections occurred in April 2013. The diarrheal frequency (times/d) and WBC counts of the infected outpatient group with NoVs were significantly higher than were those of the uninfected outpatient group.

**Conclusion:**

NoVs were confirmed to be the major viral agents responsible for acute gastroenteritis in outpatient children in Huzhou, China, and GII.4/Sydney and GII.4/2006b variants were identified as the predominant strains in this study.

## Introduction

Acute gastroenteritis (AGE), which is characterized by the onset of acute diarrhea with or without vomiting, is one of the most common infectious diseases in humans, particularly in young children aged less than 5 years of age and causes substantial morbidity and mortality in developing and developed countries[1–3]. Of children aged <5 years, more than 2.5 billion children are estimated to be infected with acute gastroenteritis, with approximately 1.5 million deaths occurring worldwide annually[4–6]. The incidence of AGE caused suddenly by viral, bacterial, and parasitic enteropathogens has steadily decreased with improvements in sanitation levels and lifestyles; however, viral agents are responsible for more than 50% of all AGE cases[2,6–8]. Enteric viruses, including group A rotavirus (GAR), norovirus (NoV), sapovirus (SaV), astrovirus (AstV), and enteric adenovirus (EAdV), are the primary etiological agents of viral gastroenteritis with acute diarrhea[9,10].

Human NoVs are recognized as the leading causes of sporadic cases and outbreaks of acute viral gastroenteritis across all age groups worldwide[11–13]. Systematic reviews have suggested that NoVs generally cause a mild and self-limiting disease, and typical clinical manifestations of NoVs include the sudden onset of watery diarrhea, fever, nausea, vomiting and abdominal cramps. However, NoVs are also significantly associated with severe gastroenteritis, causing an estimated 200,000 deaths annually in young children under 5 years of age, and large outbreaks of NoV gastroenteritis often cause substantial health burdens[14–17].

NoVs are single-stranded, positive-sense RNA viruses that belong to the genus *Norovirus* of the *Caliciviridae* family. NoVs are classified into five genogroups (GI-GV), of which GI, GII and GIV include over 30 genotypes are commonly associated with infection in humans[18,19]. The NoVs of GII are more predominant than those of GI and GIV; in particular, GII.4 (genogroup II, genotype 4) variants in recent years are the prevalent genotype responsible for viral gastroenteritis epidemics in many countries[18–21]. NoVs are genetically highly variable; thus, constant surveillance is critical for acquiring the clinical and molecular characterization of NoV infections, which is helpful for preventing and controlling NoV infections. Our previous surveillance indicated that NoVs were important agents of viral gastroenteritis in outpatient children in 2010[2]. The present study was performed to evaluate the incidence rate, age distribution, seasonal trends, and clinical and molecular characteristics of NoV infections among outpatient children with acute gastroenteritis in Huzhou, China, from April 2013 to April 2014.

## Materials and Methods

### Ethics statement

According to the Declaration of Helsinki (1964), this study was approved by the ethics committee of the First People’s Hospital of Huzhou Teachers College and Huzhou Central Hospital, and signed informed consent was obtained from the parents or legal guardians of all subjects.

### Patients and samples

Five professional nurses and five physicians collected stool samples from children under 5 years of age with acute gastroenteritis that were treated from April 2013 to April 2014 at the Outpatient Department of Pediatrics of the First People’s Hospital of Huzhou Teachers College and at Huzhou Central Hospital. A definition of acute gastroenteritis is described in a previous report[2]. Briefly, patients with acute gastroenteritis present watery and/or loose stools with ≥3 instances within a 24-hour period. Additionally, 143 age- and gender-matched healthy children without AGE were selected from health checks at our hospital that required stool and peripheral blood samples to test for pathogens including parasites, GAR, EAdV and NoV, peripheral blood cells, or HBV infection status upon the parents’ request or physicians’ advice and were included in our study.

One fecal sample was collected from each child, and these specimens were stored at -80°C until analyzed for NoVs. In addition, data regarding age, clinical manifestations and other information were recorded during face-to-face interviews in this study.

### Nucleic acid extraction and norovirus detection

All stool samples were diluted to 10% suspensions in phosphate-buffered saline (PBS, pH 7.4) and clarified by centrifugation at 8,000×g for two minutes. Nucleic acids were extracted from 140 μL of the fecal suspensions using a QIAamp Viral RNA Mini Kit (Qiagen, Hilden, Germany) according to the manufacturer’s instructions. The cDNA of the viral RNA was generated by reverse transcription using a PrimeScript RT Kit (TaKaRa, Dalian, China) with random primers according to the manufacturer’s protocol. PCR was performed for the detection of GI and GII NoVs using previously described primers[2]. The final volume for each reaction was 50 μL. Each reaction contained 5.0 μL of 10× Ex Taq DNA polymerase buffer; 4.0 μL of 2.5 μM dNTPs; 0.5 μL of mixed primers for GI-SKF, GI-SKR, CoG2F and G2SKR (10 μM each); 0.25 μL of ExTaq DNA polymerase (5 U/μL), 5 μL of cDNA, and 35.25 μL of nuclease-free water. The amplification conditions were set as follows: 94°C for 5 min, followed by 35 cycles of 94°C for 30 s, 55°C for 30 s, 72°C for 1 min, and an extension step at 72°C for 7 min. All PCR products were analyzed by 2% agarose gel electrophoresis.

### DNA sequencing and phylogenetic analysis

Positive PCR products were randomly selected, purified and sequenced using an ABI 3730 XL DNA analyzer (Applied Biosystems, Foster City, CA) of Sangon Biotech Co., Ltd. (Shanghai, China). The nucleotide sequences were compared with those of NoV strains from the GenBank database. The phylogenetic relationships of the NoVs were analyzed by aligning sequences using ClustalW software, and phylogenetic trees were constructed by the maximum likelihood model method using MEGA version 4.0 software (Tamura, Dudley, Nei, and Kumar 2007)[22]. Significant difference between inferred phylogenies was analyzed by bootstrap analysis with 1000 pseudoreplicate data sets. All nucleotide sequences from this study were deposited in GenBank with accession numbers KR047106-KR047116 for GI genotype of NoV (11 capsid sequences) and KR047117- KR047181 for GII genotype of NoV (65 capsid sequences).

### Statistical analysis

Statistical analysis was performed with SPSS software (version 13). Chi-square (χ^2^) tests and Student’s *t*-tests were performed, and the results were considered statistically significant at *P*<0.05.

## Results

### NoVs detection

Of 1346 stool specimens collected from outpatient children under five years old with AGE from 2013 to 2014, 28.5% (383/1346) were NoV positive, including 1.6% with GI genotypes (21/1346) and 26.9% with GII genotypes (362/1236). The male to female ratio was 1:0.8 (213/170) for norovirus infections included 12 males and 9 females with GI genotypes and 201 males and 161 females with GII genotypes (Table 1). These enrolled patients were designated as patients infected with NoV, thus the remaining 963 individuals with AGE were considered uninfected patients (Table 2). Additionally, no NoV subtypes were detected in the stools from the enrolled healthy children.

**Table 1:** Detection of norovirus with GI and GII genotypes in outpatient children with acute diarrhea.

Gender	No. of cases	NoV cases (%)	NoV-GI cases (%)	NoV-GII cases (%)	χ2^a^	*P* value
	1346	383 (28.5)	21 (1.6)	362 (26.9)	353.97	<0.001
Male	722	213 (29.5)	12 (1.7)	201 (27.8)	196.72	<0.001
0030	624	170 (27.2)	9 (1.4)	161 (25.8)	157.34	<0.001
χ2^b^		0.838	0.105	0.707		
*P* value		0.36	0.746	0.4		

^a^ GI cases vs. GII cases.

^b^ Male cases vs. female cases.

**Table 2:** Comparison of clinical manifestations of outpatients between NoV infections and no-NoV infection.

Clinical manifestations	Outpatients with NoV infections (n = 383)	Outpatients without NoV infections (n = 963)	*P* value
Vomiting	135 (35.2%)	325 (33.7%)	0.274
Diarrhea frequency (times/d)	5±1	4±1	<0.0001
Fever (>37.5°C)	104 (27.2%)	358 (37.2%)	12.208
WBC (×10^9^/L)	9.9±4.0	9.3±3.5	0.009
Leukocytes	4.1± 2.0	4.2±2.0	0.852
Neutrophils	4.9±3.2	4.4±3.0	0.009

The values are presented as the mean ± standard deviation (SD). Additionally, the referenced denominator of children evaluated for WBC, leukocyte and neutrophil counts were 4.0–12.0, 2.0–7.2 and 1.5–6.0 (×10^9^/L), respectively.

### Clinical manifestations of children with NoV infections

The clinical manifestations of the children with NoV infections are shown in Table 2. The percentage of the cases with vomiting or fevers (>37.5°C) did not differ between the infected and uninfected patients with NoVs (*P*>0.05). However, the diarrheal frequency (times/d) of the patients with NoV infections was significantly higher than that of the patients without NoV infections (5.2±1.2 vs. 4.2±1.0, respectively, *P*<0.0001). In addition, the white blood cell (WBC) counts in peripheral blood samples (9.9±4.0×10^9^/L) from the patients with NoV infections increased compared to that of the patients without NoV infections (9.3±3.5×10^9^/L), and this difference was statistically significant (*p* = 0.009). The neutrophils of WBC counts were elevated in the patients with NoV infections compared to the patients without NoV infections, but the difference was not statistically significant (4.1±2.0 vs. 4.2±2.0, respectively, *p* = 0.852). However, the leukocytes of WBC counts in peripheral blood samples from the patients without NoV infections were significantly higher than those of the patients with NoV infections (4.9±3.2 vs. 4.4±3.0, respectively, *p* = 0.009). Additionally, in the 143 healthy children, no fevers were >37.5°C, the pathogens of parasites, GAR, EAdV and NoV were not detected, and the counts of WBCs, neutrophils and leukocytes were 6.4±1.5, 4.2±1.9 and 2.2±1.7 (×10^9^/L), respectively.

### Age and seasonal distribution of children with NoV infections

**Fig 1:**
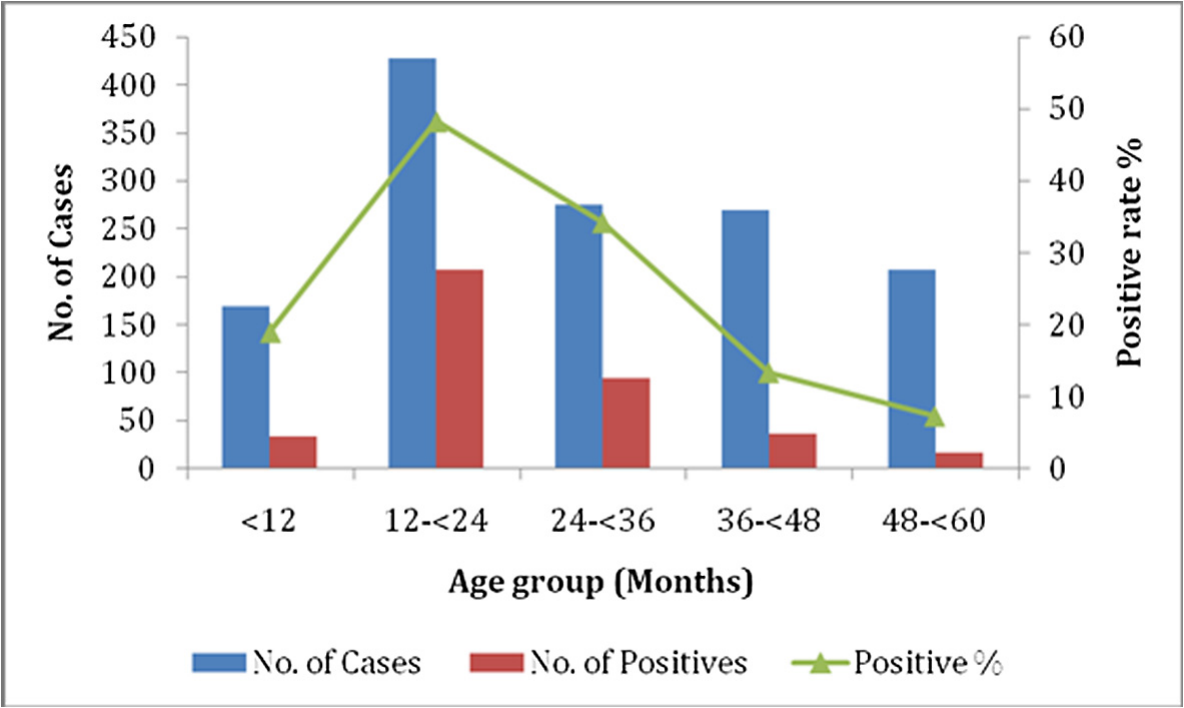
Age distribution (months) of outpatient children with acute diarrhea caused by norovirus infections.

**Fig 2:**
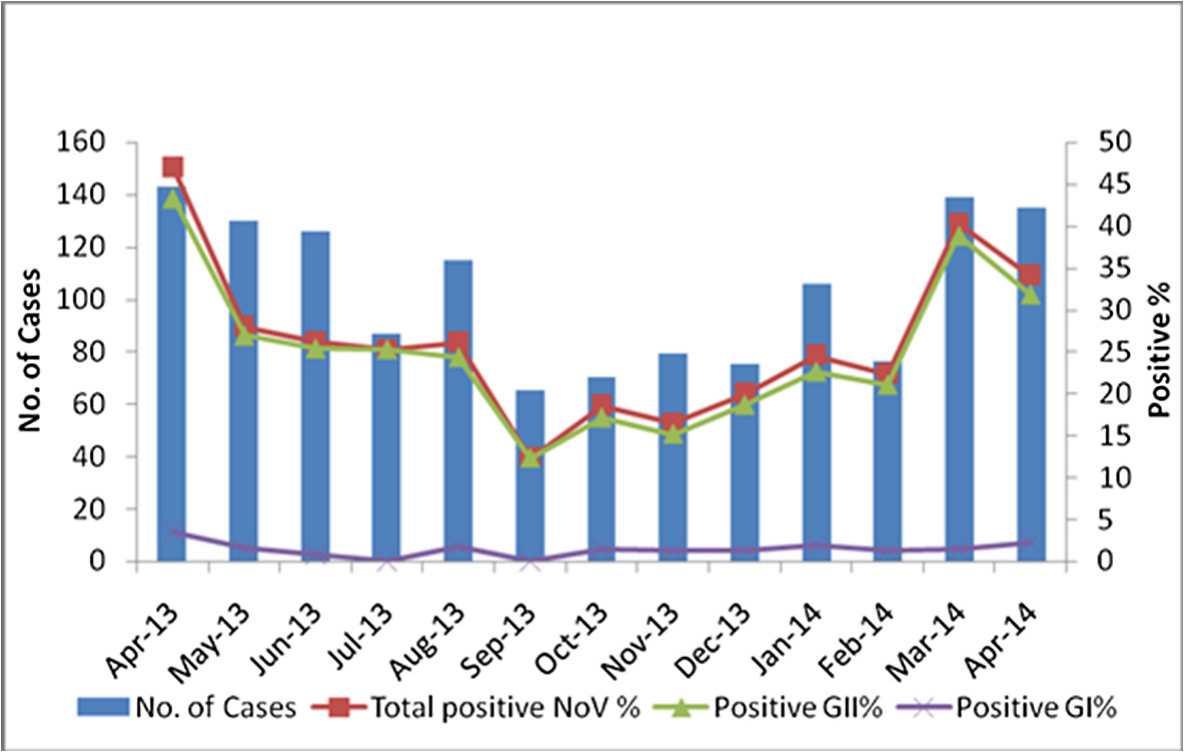
Seasonal distribution (months) of outpatient children with acute diarrhea caused by norovirus infections.

In this study, NoV infections were the most common in the 12–24 month age group (48.2%) compared with the other age groups (7.3–34.2%). For the 383 children with NoV infections, the prevalence rate was up to 86.7% (332/383) in children under 3 years of age, which was significantly higher than that of children above 3 years of age (13.3%, 51/383) (χ2 = 68.01, *p<0*.*001*) (Fig 1). NoV infections occurred commonly throughout the year, and the infection peak occurred in the spring of 2013 and 2014 (Fig 2), with the highest proportion occurring in April 2013 (46.9%, 67/143).

### NoV genotype analyses

**Fig 3:**
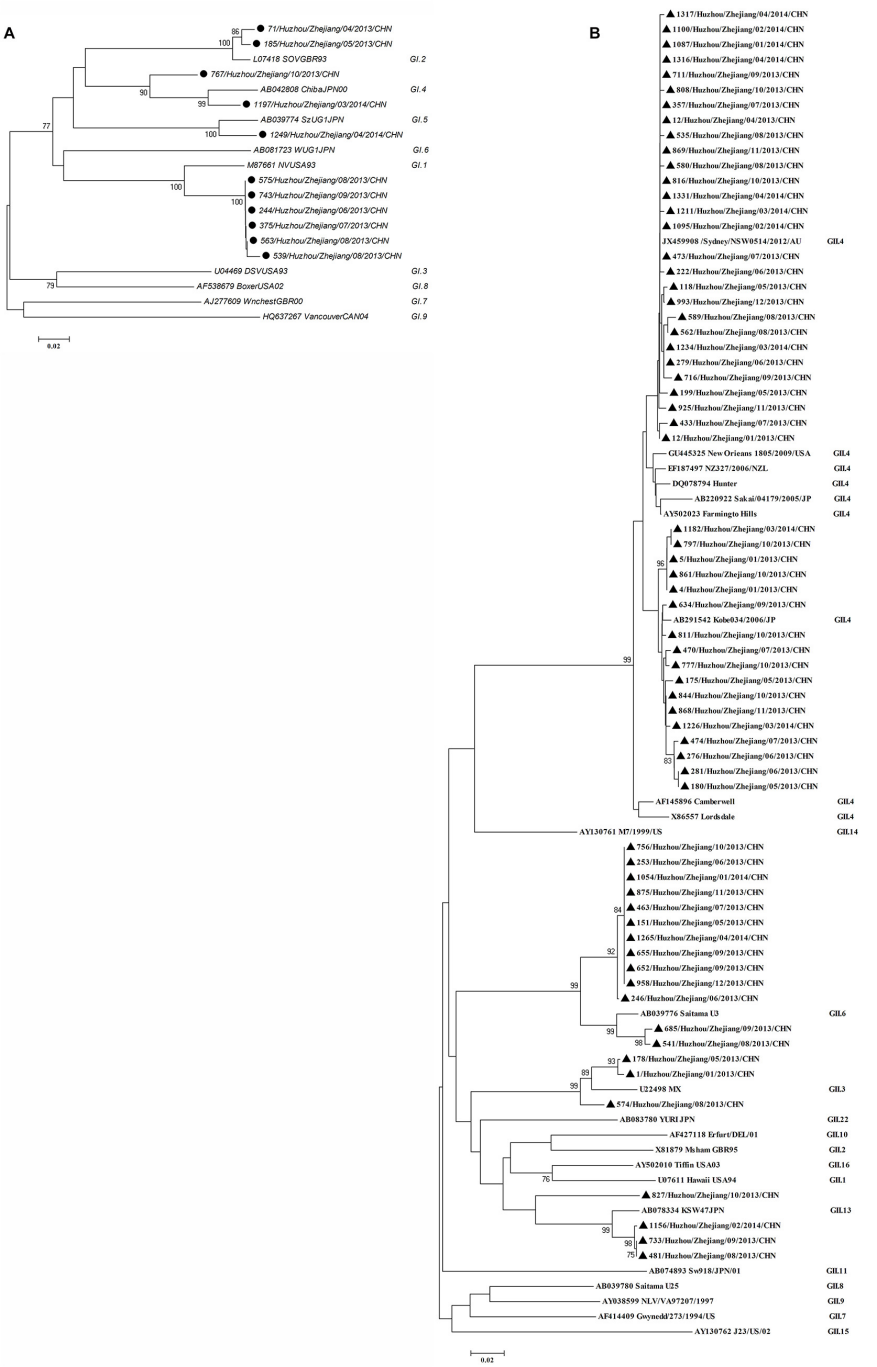
Phylogenetic analysis of the partial capsids of the NoV strains. The phylogenetic trees were constructed by the maximum likelihood model method. The results are shown for the GI (A) and GII (B) genotypes. ●, represents the GI genotypes in this study; ▲, represents the GII genotypes in this study.

A phylogenetic tree of nucleotide sequences of the partial capsid region of NoV GI or GII isolates was constructed compared with the nucleotide sequences of the reference strains in the GenBank database (Fig 3). Eleven GI NoV strains were clustered into 4 genotypes that included 6 GI.1, 2 GI.2, 2 GI.4 and 1 GI.5 strains (Fig 3A). Among 65 NoV strains, the GII.4 genotype was the predominant strain (69.2%, 45/65), along with three other genotypes that included 3 GII.3, 13 GII.6, and 4 GII.13 strains. The GII.4 genotype clustered into the GII.4/2006b (37.8%, 17/45) variant and the GII.4/Sydney variant (62.2%, 28/45) (Fig 3B).

## Discussion

NoVs are common causative agents of acute gastroenteritis in young children under 5 years of age and have caused a series of outbreaks worldwide[2,15]. Viral gastroenteritis caused by NoVs remains a public health burden to humans in all age groups in developing and developed countries[1,6]. However, few reports have examined the prevalence of NoVs in outpatient children < 5 years old with AGE in developing countries. In the current study, NoVs were detected in 28.5% (383/1346) of the fecal specimens collected from outpatient children with acute diarrhea. The detection rate is similar to previously reported detection rates, which range from 3.5% to 47.3%[23,24]. However, the detection rate of NoVs in the children in this study was clearly higher than those that have been previously reported for children in some countries (18.1% in southeast China, 13.2% in Japan, 15.0% in Korea, 20.4% in Brazil and 7.4% in America)[2,25–28]. This discrepancy might be due to varying study periods and poor sanitation, as well as the geographical distribution of the studied population.

Some important clinical features such as diarrhea, vomiting, and fever have been reported in NoV gastroenteritis[29,30]. Chen et al. reported that the frequency of diarrhea was 4.3±1.9 times/day and that the percentage of vomiting was 27.5% in outpatient children[2]. Sai et al. reported that vomiting, fever ≥37.5°C, and diarrhea frequency were 65.5%, 19.0%, and 4.6±1.1 episodes/day, respectively, in outpatient children[31]. In the current study, the frequency of acute diarrhea in patients with NoV infection was significantly higher than that of patients without NoV infections (5.2±1.2 vs. 4.2±1.0, respectively, *p*<0.0001). However, the proportion of vomiting was not significantly different between the children with NoV infections (35.2%) and children without NoV infections (33.7%) (*p*>0.05), which was similar to the percentages the fevers (>37.5°C) between the children with NoV infections (27.2%) and the children without NoV infections (37.2%) (*p*>0.05). These results were slightly different from those of other studies[2,31,32], which might be associated with the differences in age distribution, study periods and study population, i.e., with or without hospitalized children. Additionally, WBCs counts and the neutrophils in peripheral blood samples from the patients without NoV infections were significantly low, but the leukocytes in peripheral blood samples from these patients were slightly high compared to the patients with NoV infections, which were consistent with a previous report[32]. These discrepancies might be due to differences in the age distribution of the patients enrolled in the studies.

NoV infections occurred in all age groups. Recently, in sporadic cases of viral gastroenteritis, NoV was the second most common pathogen detected and was only lower than GAR in children under five years of age[1,2,4,6,31–33]. In our study, the peak detection rate of NoV infection occurred in patients 12–24 months of age, and the percentage of positive cases of NoV infections was up to 86.7% of all positive patients with NoV infections under three years of age. Thus, NoVs easily infect children under three years of age, particularly those children 12–24 months of age. These findings indicated that the age of children was closely associated with the rate of NoV infections and that this peak might be due to poor patient hygiene and/or to frequent touch with poor sanitation around them because children 12–24 months of age may have more opportunities to be exposed to NoV-infected environments than may children of other age groups. Moreover, the weak immune systems of younger children might contribute to their higher susceptibility to NoVs than that of older children. Many studies have indicated that NoV infections are affected by seasonal distribution and occurs commonly in the spring, summer, and winter[2,30,33]. In Poland, Malawi, and Argentina, NoV infections were easily detected in the spring and/or summer[34,35]. However, in Japan and Canada, NoV infections occurred commonly in the winter[36,37]. In this study, sporadic NoV infections occurred more commonly in the spring, with the peak detection rate in April 2013, similar to the results reported for southeastern China[2]. These results indicated that the geographical diversity of the surveillance areas along Tai Lake likely affected the variability in the NoVs seasonal trend. Moreover, the warm and wet climate conditions in the monitored area were important agents of NoV infections.

Many molecular epidemiological surveys for NoVs have demonstrated that NoVs are one of the most pathogenic causes of sporadic and epidemic diarrhea, and GII genotypes of NoVs are known to have a wider circulation than GI genotypes in China and other countries[6,26–29]. Although the GI and GII genotypes have diversity, the GII.4 genotype is the predominant strain that causes global gastroenteritis oubreaks[18–21]. In our study, the genetic diversity of GI NoVs was observed in 6 GI.1, 2 GI.2, 2 GI.4, and 1 GI.5 strains, and the GII.4/Sydney variants were the primary strain, which is consistent with previous studies[20,30]. Moreover, the second cluster strain was the GII.4/2006b variant, which is the most predominant strain that has been circulating in both children and adults with acute diarrhea in China since 2006[20]. These results indicated that the GII.4/Sydney variant and GII.4/2006b variant were more important strains than other genotypes that were associated with acute diarrhea in children in Huzhou, China. The differences from previous surveys might be related to the poor environment and geographical distribution of the monitored area, as well as to host factors, climate and human behavior, such as diet[4,6].

In conclusion, our data indicated that the predominant GII.4/Sydney variant and GII.4/2006b variant simultaneously occurred in Huzhou, China. These variants are associated with an increase in NoV epidemics worldwide. Therefore, persistent surveillance for NoV infections in children with acute diarrhea should be emphasized to prevent large-scale epidemics of NoVs and to reduce the health burden to humans.
